# Assessment of Mercury Intake from Fish Meals Based on Intervention Research in the Polish Subpopulation

**DOI:** 10.1007/s12011-017-0939-9

**Published:** 2017-01-27

**Authors:** Renata Kuras, Beata Janasik, Magdalena Stanislawska, Lucyna Kozlowska, Wojciech Wasowicz

**Affiliations:** 1 Department of Biological and Environmental Monitoring, Nofer Institute of Occupational Medicine, 8 Teresy St, 91-348 Lodz, Poland; 2 Department of Dietetics, Faculty of Human Nutrition and Consumer Sciences, Warsaw University of Life Sciences, Warsaw, Poland

**Keywords:** Mercury, Environmental exposure, Fish consumption, PTWI, TDA-AAS

## Abstract

The paper’s objective was to estimate weekly Hg intake from fish meals based on intervention research. Total Hg (THg) concentrations in blood and hair samples collected from men (*n* = 67) from an intervention study as well as muscular tissues of fresh and after heat-treating fish were determined using the thermal decomposition amalgamation atomic absorption spectrometry method (TDA-AAS) using direct mercury analyzer (DMA-80). The mean of the estimated weekly intake (EWI) was estimated at 0.62 μg/kg bw/week in the range 0.36–0.96 μg/kg body weight (bw) /week through the consumption of 4 edible marine fish species every day (for 10 days) by the participants from the intervention research in Lodz, Poland. The Hg intake in the volunteers in our intervention study accounted for 38.6% of the provisional tolerable weekly intake (PTWI) (1.6 μg/kg bw, weekly) value. The average Hg concentration in the analyzed fish ranged from 0.018 ± 0.006 mg/kg wet weight (*Gadus chalcogrammus*) to 0.105 ± 0.015 mg/kg wet weight (*Macruronus magellanicus*)*.* The results for the average consumers were within PTWI of methylmercury (MeHg). Moreover, the average concentration of Hg in the selected fish after heat treatment did not exceed the maximum permitted concentrations for MeHg (MPCs = 0.5 mg/kg wet weight) in food set by the European Commission Regulation (EC/1881/2006). Hence, the risk of adverse effects of MeHg for the participants is substantially low.

## Introduction

Food contamination is an issue that still raises attention. Harmful side effects for human health resulting from the intake of food contaminated with chemical substances have been the most frequently observed ones [1]. One of neurotoxicants that have received most attention with this respect is methylmercury (MeHg). The risk assessment for MeHg is based primarily on the data obtained in the past, i.e., on epidemiological studies (large-scale), which related to fish consumption among pregnant/breastfeeding mothers/children [2].

Humans are exposed to organic forms of mercury through fish and seafood consumption, in particular top predatory fish such as swordfish, marlin, king mackerel, tile fish, shark, and tuna. Fish may accumulate MeHg directly from water (first Hg in water is converted by sulfate-reducing bacteria into an organic form) or through consuming other organisms (biomagnification of the food chain). Total Hg concentration in fish is often used as a measure of MeHg exposure, assuming that almost 100% Hg concluded in fish and seafood occurs in MeHg [3]. In the gastrointestinal tract, MeHg is absorbed to an extent of about 95%. Its lipophilic properties facilitate a smooth transition between the blood-brain barrier (BBB) as well as placental barrier, which impairs metabolism of the nervous system. Mercury compounds, both organic and inorganic, are extremely toxic due to the high affinity with thiol groups of enzymes and proteins. Ingestion of fish contaminated with MeHg can lead to adverse health outcomes (ataxia, paresthesia, dysarthria, hearing defects, visual disturbances) [4].

Health risks regarding consumption of Hg-contaminated fish are subject to regulation introduced by many countries and government agencies. The European Union Commission Regulation [5] sets maximum levels (MLs) only for total mercury content (THg) for fish and seafood from 0.5–1.0 mg/kg wet weight. In view of the above, the Joint Food and Agriculture Organization of the United Nations and World Health Organization FAO/WHO Expert Committee on Food Additives (JECFA) has established provisional tolerable weekly intake (PTWI) for MeHg as the amount of contaminant (e.g., MeHg), which is not cleared rapidly from the body and that can be ingested over a lifetime without appreciable risk. The PTWI value is calculated for a week due to the bioaccumulation of the contaminants (e.g., toxic elements) in humans.

The PTWI for MeHg of 1.6 μg/kg bw/week corresponds to 0.112 mg/week for a person weighing 70 kg. The PTWI for THg is 4 μg/kg bw/week [6, 7]. The (US) National Research Council (NRC) established an intake limit of 0.7 μg/kg bw/week, the 2.3-fold lower limit than that of JECFA [4].

The estimated Hg intakes are different between the European countries and depend on the amount and the type of fish consumed. In Poland, the PTWI value based on the European Food Safety Authority (ESFA) [5, 8] is in line with the JECFA and concerns Hg and MeHg in food.

Assessment of exposure to MeHg can be carried out on the basis of measurements of Hg in food (fish and fishery product) or based on the results of biological monitoring of Hg concentrations in blood (Hg-B) and hair (Hg-H). Hg-B, in particular Hg-H, are routinely used as biomarkers to assess MeHg exposure [9, 10]. Until recently, to determine mercury content in biological materials, various methods of digestion-utilizing chemical reagents (strong bases or acids) or in combination with microwave mineralization were used [11, 12]. The latest method is measurement by means of cold vapor atomic absorption spectrometry (CVAAS) [13] or by inductively coupled plasma mass spectrophotometer (ICP-MS) after microwave-assisted acid digestion [14]. However, the simplest and most effective method of Hg determination, where sample preparation or other wet chemistry is not required before the analysis, is the direct mercury analyzer DMA-80 [15, 16].

In order to estimate the dietary intake of chemical substances, chemical analysis and appropriate estimation methods are used. We can choose one of the three variants of contamination levels: the most probable case (estimation of single or short-period intake, for instance over a week), the average case (for a long period), and the extremely high case—mostly 90th percentile of the distribution of concentrations or an average of 10% of the results of the highest value. The last is connected with consuming food from one source, mostly from heavily industrialized areas. In this paper, the authors chose the first option. Estimation of dietary exposure to MeHg in fish was based on the assumption that almost 90% of the total Hg that is present in fish meat, fish products, fish offal, and seafood exists in the form of MeHg [8]. In order to assess the risk of adverse health effects among individuals who consume more fish meals than average, it is necessary to estimate the intake of heavy metals contaminating this food.

The main objective of this study was to estimate whether the PTWI value of MeHg for men in Poland is safe. The projected intake values of Hg through human consumption were calculated (microgram per kilogram body weight (bw) weekly) and compared with the PTWI value as stipulated by the JECFA/ESFA. Moreover, this paper examined variations in Hg concentrations in blood and hair as biomarkers of MeHg intake correlated with fish consumption as well as Hg levels in fish commonly eaten by the Polish subpopulation.

## Materials and Method

### Study Design and Participants

An intervention study, which was undertaken between June and August 2015, involved 71 healthy men from the Nofer Institute of Occupational Medicine (NIOM) in Lodz, Poland. The participants were recruited via the information posted in NIOM and were informed about the aim of the study and of the examinations to be performed and then signed informed consent forms. In order to obtain the basic data needed for the research, personal survey and the food frequency questionnaire (FFQ) were conducted. The survey included questions about age, body mass index (BMI), current smoking status, diet (including intake of fish oil or supplements containing shark cartilage), alcohol, and medical history. Four individuals were rejected from the study due to fish oil and omega-3 fatty acid supplementations. The study men were aged 21–64 years with a mean age of 41 years. Their BMI was 26.9 ± 4.3 kg/m^2^ in the range of 17.8–40.2 kg/m^2^. The 21 % of the subjects were current smokers. The 34 % had from one to five dental amalgam fillings. The FFQ showed that the diet of the study individuals was similar. The FFQ provided us with details concerning fish consumption among the individuals. According to the FFQ, 9% of the volunteers never (or almost never) ate fat fish (e.g., salmon, sardines, herring, mackerel, big carp, eel), 50% ate such fish once a month or more seldom, and 40% several times a month. The lean fish (e.g., pollock, cod, perch, hake, carp, small tuna, pangasius, trout) consumption presents as follows: never or almost never—4%, once a month or less—52%, and several times a month—41%. None of the study subjects ate supplements containing shark cartilage or fish oil. Seventy-six percent of the individuals never or almost never ate game meat, 20% once a month, or more seldom.

The types of fish were selected based on the market analysis of fish consumption in Poland (Institute of Agricultural and Food Economics National Research Institute (IAFE-NRI), data from the year 2014). According to the data of the IAFE-NRI, in Poland, in 2013 and 2014, the biggest increase in fish consumption per one resident in the total consumption of fish concerned salmon and cod, while the Polish consumers most frequently ate pollock (up to 25% of the chosen fish). Of eight initially selected fish, four marine fish species of the family Gadiformes with higher concentration of Hg were selected. Patagonian (*Macruronus magellanicus* other name *Patagonian grenadier*), pollock (*Gadus chalcogrammus*), cod (*Gadus morhua*), and coalfish (*Pollachius virens*) came in a form of frozen filet samples (without skin) from the Polish market. The fish species were caught in the zone of the Pacific Ocean in fishing area FAO 87 (*M*. *magellanicus*) and FAO 61 (*G*. *chalcogrammus*) and in the area north-east Atlantic FAO 27 *(G*. *morhua*, *P*. *virens*).

The intervention research consisted in daily fish consumption (frying fish dishes) for 10 days (2 weeks with the exception of Saturdays and Sundays). The volunteers ate on three consecutive days *M*. *magellanicus* (the first week) and *G*. *morhua* (the second week) and on two consecutive days *G*. *chalcogrammus* (the first week) and *P*. *virens* (the second week). During the whole study, all the participants consumed approximately 1.6 kg of fried fish, which was equivalent of about 0.16 ± 0.006 kg of fried fish daily. Study participants ate fish in a canteen of the NIOM. Each portion of fish (without spices and butter) was weighted before and after frying. The fish was fried (common frying temperatures 170–190 °C) in rapeseed oil from 5 to 10 min. With the purpose of monitoring Hg concentrations in blood and hair, the samples were collected within certain intervals. On the first day (named “test I”) of fish consumption, blood and hair samples were collected and the FFQ questionnaire was carried out. Then, after 1 week (named “test II”) and at the end of the second week of fish consumption (named “test III”), blood samples were collected. Finally, 1 month after the study (named “test IV”), blood and hair samples were collected once again. The study was approved by the Ethics Committee of the Nofer Institute of Occupational Medicine in Lodz, Poland.

### Specimen Collection

Blood was collected into a *Venosafe* tube (free from trace and heavy metals) containing Lithium Heparin as anticoagulant and stored at −20 °C until analysis.

For determination of Hg in hair, about 10 mg of hair samples with 1-cm length was collected. The hair was cut with scissors from the occipital area of the head at the hair root and placed in a polyethylene bag and stored at room temperature until the analysis. Due to the fact that all the participants were men, the hair samples were only rinsed with acetone and deionized water (18-MW). The hair washing procedure is recommended by the International Atomic Energy Agency (IAEA) [17]. We applied no additional washing method assuming that none of the volunteers used hair sprays, mousses, gels, and oils.

### Direct Mercury Analyzer DMA-80

Total mercury concentrations of muscular tissues of fish (fresh and after heat treating—frying) as well as hair and blood samples collected from the men were determined on sample boats using the thermal decomposition amalgamation atomic absorption spectrometry method (TDA-AAS) (direct wercury analyzer DMA-80 by Milestone, Spectro-Lab, Poland). The working principle of the instrument is based on the thermal decomposition, catalytic conversion, amalgamation, and atomic absorption spectrophotometry. The absorption intensity was measured at 253.7 nm. The decomposition products are carried by continuous flow of pure oxygen through a catalyst bed, where interferences are eliminated.

The analytical balance Sartorius Quintix model (Q224 1CEU) with internal calibration was used to weigh the hair and fish samples using units of milligrams.

The linear calibration curve in the range 0.5–10.0 ng was plotted as absolute mass of Hg (nanogram) versus absorption peak area. The correlation coefficient *r* = 0.9998 was achieved. *D*_L_ and *Q*_L_ were counted as 3 times (D_L_ 3 s _for blank_) and 6 times (Q_L_ 6 s _for blank_) of standard deviation for blank and were 0.0025 ng and 0.0051 ng, respectively.

Accuracy of the method was checked by the regular use of the certified reference material: DOLT-5 (fish liver, National Research Council of Canada, NRC—CNRC) and standard reference material: NIST 2976 (SRM—Mussel Tissue, Trace Elements and Methylmercury, Freeze-dried). To check determination of mercury in hair, we used human hair powder reference material: NCS DC 74337 (Certified Reference Material, China National Analysis Center for Iron and Steel, China).

Seronorm trace element whole blood reference material (RM) as well as blood samples from the international program of the German External Quality Control (G-EQUAS) was used for the method development and validation. All the results of the analyzed (certified) reference materials had satisfactory recoveries from 95% (DOLT-5) to 107% (NCS DC 73347a).

To prepare standard solutions containing mercury we used an inorganic mercury standard stock solution (1000 μg Hg/ml in 2% HNO3; JT Baker), hydrochloric acid (36.5–38% HCl; JT Baker), and deionized water (resistivity of 18 MΩcm) in the Milli-Q Integral 3 system (Millipore, Bedford, MA, USA). The blood was collected into Venosafe tube containing Lithium Heparin (VF-054SHL).

### Statistical Analysis

Statistical analysis was conducted using Statistica 8.0 sofware (StatSoft Inc., Poland). Significance was established at the level of *p* ≤ 0.05. We tested deviation from the Hardy-Weinberg equilibrium using the chi-square test. The Shapiro-Wilk test was used to determine normality of distribution [18, 19].

## Results

### Mercury in Fish

The average concentration of Hg in muscle tissues of fish is presented in Table 1. The average Hg concentration in the analyzed fish ranged from 0.018 ± 0.006 mg/kg wet weight and 0.023 ± 0.006 mg/kg wet weight (*G*. *chalcogrammus*) to 0.105 ± 0.015 mg/kg wet weight and 0.109 ± 0.015 mg/kg wet weight (*M*. *magellanicus*), respectively, in raw and fried fish and decreased (both in a raw and fried state) as follows: *M*. *magellanicus* > *G*. *morhua* > *P*. *virens* > *G*. *chalcogrammus*. Fried fish contained slightly higher amount of Hg than raw fish with a mean of 0.058 ± 0.033 mg/kg (median = 0.053 and 95th percentile = 0.115) and 0.054 ± 0.034 mg/kg (median 0.044 and 95th percentile 0.119), respectively. The differences between Hg level in raw and fried fish species were from 0.4% for *M*. *magellanicus*, 0.5% for *G*. *chalcogrammus*, 1.1% for *G*. *morhua* to 1.4% for *P*. *virens.*

**Table 1:** Mean and weight expressed as arithmetic mean (standard deviation, SD), range, median, selected percentile P95 (95% confidence intervals CI) concentrations of Hg in muscle of fish species (milligram per kilogram) and weight of fish (kilogram) (*n* = 12)

		Raw fish					After heat treatment				
Fish species	Academic name	Mean Hg (SD)	Hg range	Median	P95	Fish weight (SD)	Mean Hg (SD)	Hg range	Median	P95	Fish weight (SD)
Hoki, Patagonian	*Macruronus magellanicus*	0.105 (0.015)	0.075–0.130	0.11	0.12	0.175 (0.015)	0.109 (0.015)	0.052–0.139	0.11	0.13	0.150 (0.014)
Pollock	*Gadus chalcogrammus*	0.018 (0.006)	0.010–0.028	0.02	0.03	0.166 (0.020)	0.023 (0.009)	0.009–0.033	0.02	0.03	0.147 (0.019)
Cod, salt cod, codfish	*Gadus morhua*	0.049 (0.006)	0.041–0.060	0.05	0.06	0.189 (0.025)	0.060 (0.009)	0.049–0.072	0.06	0.07	0.173 (0.026)
Coalfish, saithe, coley, sablefish	*Pollachius virens*	0.041 (0.005)	0.027–0.046	0.04	0.05	0.189 (0.023)	0.055 (0.019)	0.035–0.090	0.05	0.09	0.166 (0.021)

Moreover, average concentration of Hg in selected fish after heat treatment did not exceed the maximum permitted concentrations (MPCs =0.5 mg/kg) in food set by the commission regulation [5].

### Blood-Hair Relationship

**Fig. 1:**
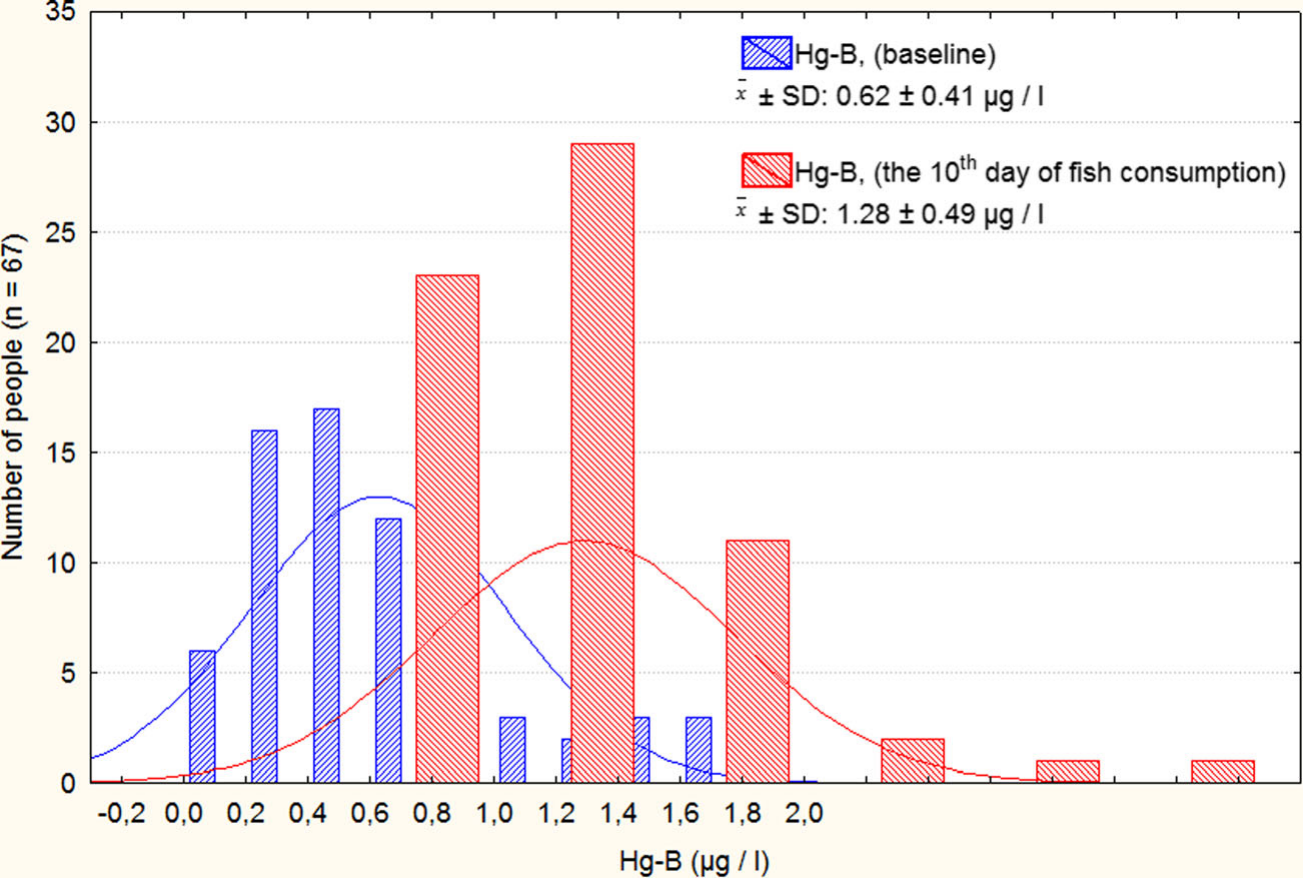
The distribution of THg concentration in whole blood samples measured in 67 men during intervention

The blood samples were taken before dosing and during the test, then they were analyzed for mercury concentrations. Mean concentration of Hg in the whole blood (Hg-B microgram per liter) of the volunteers on the first day of fish consumption was found to be 0.62 ± 0.41 μg/l (NS); after 1 week, it was 0.90 *±* 0.46 μg/l (*p* < 0.001); in the end of the study, 1.28 *±* 0.49 μg/l (*p* < 0.001), and 1 month after the end of the study (test IV), 0.78 *±* 0.60 μg/l (*p* < 0.001). The average increase in the Hg-B concentration was from 0.62 to 1.28 μg/l. Distribution of the whole blood total Hg measured in 67 men during the intervention is presented in Fig. 1 (the first day and after 10 days of fish consumption).

**Fig. 2:**
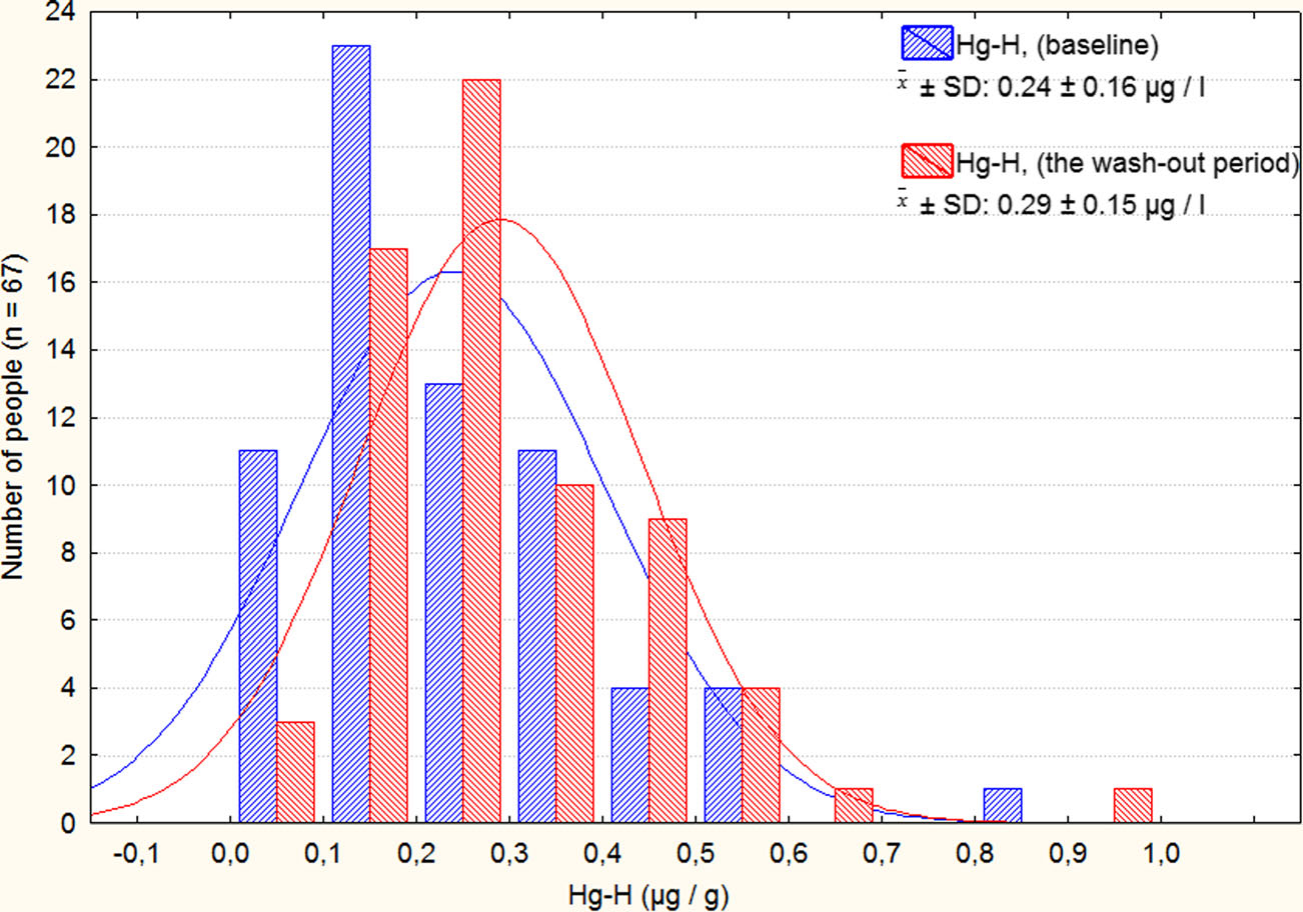
The distribution of THg concentration in hair samples measured in 67 men during intervention

Mean concentration of Hg in hair (Hg-H microgram per gram) of the volunteers on the first day of fish consumption was found to be 0.24 ± 0.16 μg/g (NS), and 1 month after the end of the study (test IV), it was 0.29 *±* 0.15 μg/g (*p* < 0.05). Distribution of total Hg in the hair of the study individuals during the intervention (the first day and 1 month after the end of the study) is presented in Fig. 2. Hg concentration in the hair depended strictly on the amount of consumed fish and the level of their pollution with mercury.

**Fig. 3:**
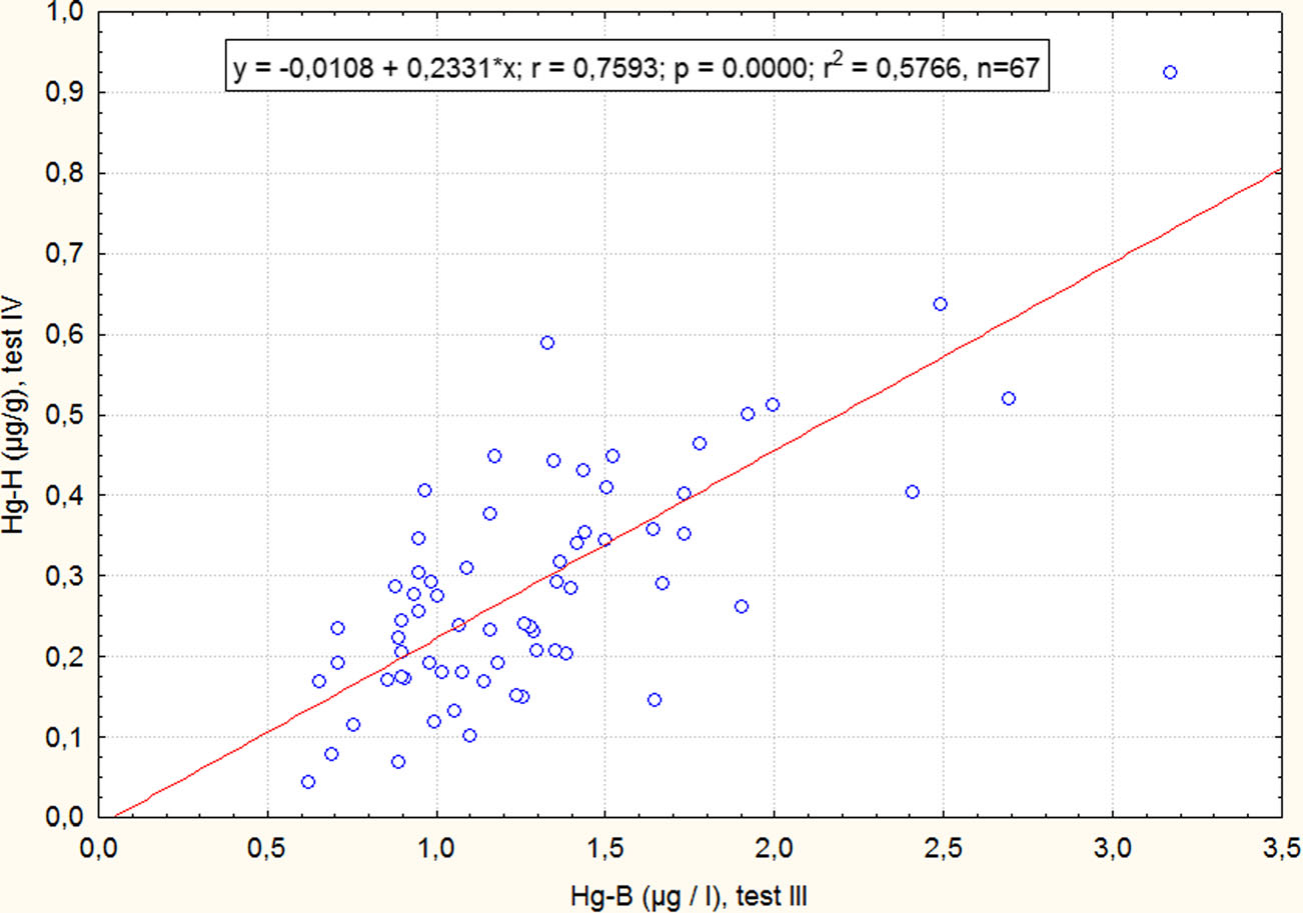
The relationship between THg-H and THg-B in 67 men evaluated by linear regression

The average Hg hair to Hg blood ratio was 0.23 (median = 0.22; 95th = 0.35; range = 0.069–0.442). Figure 3 presents concentration of mercury in the hair 1 month after the end of the study in relation to blood (Hg-B microgram per liter) collected at the end of the study (on the 10th day of fish consumption).

### Assessment of Mercury Intake from Fish

Taking into account that Hg content was expressed as microgram per kilogarm of fried fish samples and numbers of consumed fish portions, the calculated intake during 10 days of fish consumption amounted to 105 μg. The estimated daily intake of Hg in the dietary intervention study was 10.5 μg for Hg. The MeHg (microgram per kilogram) intake per kilogram of body weight per week was calculated by multiplying the amount of fish ingested per week (kilogram per week) and Hg concentrations in the ingested fish (microgram per kilogram) divided by body weight (kilogram). The estimated weekly intake (EWI) of Hg in our research, which equaled 0.62 μg/kg bw/week (median = 0.62; the range = 0.36–0.96), was within the PTWI value.

In the case of a person weighing 70 kg and consuming a meal consisting of 150 g of *M*. *magellanicus* and 173 g of *G*. *morhua* three times a week, Hg intake was higher for *M*. *magellanicus* than for *G*. *morhua* (0.70 vs. 0.45 μg/kg bw). Correspondingly, in the case of a meal consisting of 147 g of *G*. *chalcogrammus* and 166 g of *P*. *virens* two times a week, Hg intake was higher for *P*. *virens* than for *G*. *chalcogrammus* (0.26 vs. 0.09 μg/kg bw)*.* In our research project, the estimated weekly intake of Hg amounted to 0.62 μg/kg bw/week (38.6% of the PTWI), which corresponds to 0.04 mg/week for a person weighing 70 kg. The Hg intake was evaluated based on consumption of 800 g/week. The average mercury concentration in raw fish was 0.054 ± 0.034 mg/kg and the 90-percentile level of 0.12 mg/kg; thus none of the determined samples of fish exceeded the limits established in the European Union for this toxic metal (0.5 mg/kg for Hg). Fried fish contained slightly higher amount of mercury than raw fish with a mean of Hg 0.058 ± 0.033 mg/kg.

### Hg in Relation to Age, Smoking Status, and Amalgam Fillings

No significant differences in Hg concentration was found between the smokers and nonsmokers at the beginning of the study. Hg-B were found to be 0.48 ± 0.38 μg/l and 0.66 ± 0.42 μg/l (NS), respectively. Also, no significant changes in Hg-B concentration between the people with and without dental amalgam fillings was observed, i.e., (0.57 ± 0.35 μg/l and 0.63 ± 0.42 μg/l (NS), respectively. There were no statistically significant differences between the smokers and nonsmokers and between the people with and without dental amalgam fillings during the whole duration of the intervention study. Moreover, there was no statistically significant difference between the average age and Hg-B.

## Discussion

In order to evaluate ingestion-related weekly mercury exposure in humans, we compared our results with the mentioned PTWI (1.6 μg/kg bw/week) recommended by the JECFA [6]. This value was reflected in the individual food exposure. Moreover, the 50th and 95th percentile limits of estimates of average dietary exposure to Hg were also below the PTWI and amount of 0.62 μg/kg bw/week and 0.80 μg/kg bw/week, respectively. The risk index (percentage of the PTWI) amounts to 38.8% in the range = 22.7–59.8%, and the hazard index (HI) of fish consumption (the ratio of EWI to PTWI) amounts to 0.39. Despite the results, 32.8% of the volunteers exceeded the intake limit established by the US-NRC (0.7 μg/kg body weight). This intake was estimated taking into account a consumption of 800 g/week.

The tolerable daily intake (TDI) is an estimate of the average quantity of mercury from four selected fish, and it amounts to 10.5 μg Hg. When this TDI value was divided by the average body weight, the estimated TDI was 0.12 μg/kg of body weight—it was lower than the established by the JECFA value of 0.23 μg/kg of body weight [6]. The value is higher in the case of groups with high-fish consumption, e.g., TDI equals 16.3 μg Hg only for *M*. *magellanicus* 3 days per week, which corresponds with 0.19 μg/kg body weight. The average exposure to MeHg in our research project is unlikely to exceed the recommended value of PTWI; the likelihood of reaching such a PTWI level increases in the case of consumption of fish with higher Hg content. If people ate only *M*. *magellanicus* for 1 week (7 days), the EWI would be 1.34 μg/kg (84% of PTWI in the range = 49.3–130%).

Differences in Hg levels in fish before and after heat treatment (0.054 ± 0.034 mg/kg vs. 0.058 ± 0.033 mg/kg.) may be a result of dehydration process, and they depend on the species. Studies of other authors have suggested that differences concerning Hg in raw and fried fish may result from fish species [20, 21], or even from Hg pre-concentration, formation of complexes involving Hg species, and sulfhydryl groups present in tissues [22]. Nevertheless, frying selected fish species in our research project did not reduce Hg content in fish considerably.

Wojciechowska-Mazurek et al. [23, 24] have shown results from the framework of monitoring research in Poland where the average mercury contamination in fish was 0.035 mg/kg and in seafood 0.022 mg/kg and at the 90-percentile level, respectively, 0.062 mg/kg and 0.040 mg/kg. Mercury intake from fish and fishery products for a person of 60 kg was estimated at 3.2 and 5.6% PTWI. Spodniewska et al. [25] have calculated percentage of PTWI taking into consideration the mean concentration of Hg in fish from lakes of Poland and the data from the Polish Central Statistical Office (2011). The results were 3.01% PTWI of Hg and 7.89% PTWI of MeHg.

According to the SCOOP (scientific cooperation on questions relating to food) report task, the estimated intakes of mercury in Europe varied depending on a country and on the amount and the type of fish consumed [2, 8, 26]. MeHg intake (assuming that all mercury is methylmercury and that 60 kg of body weight is considered for adults) was between <0.1 μg/kg bw/week for the Netherlands, through 0.3 μg/kg bw/week for France, and 1.6 μg/kg bw/week for Portugal. The range of high exposure to THg (the 95th or 97.5th percentile as high percentile of the distribution) was estimated depending on the country and amount and equaled 0.4 μg/kg bw/week for Ireland, 1.8 μg/kg bw/week for Norway, and 2.2 μg/kg bw/week for Greece. The mean weekly consumption of fish and seafood products provided by the mentioned countries ranged from 70 g (the Netherlands), 245 g (France) to 350 g (Portugal); and for high intakes from 497 g (Greece), 525 g (Ireland) to 1925 g (Norway).

For adults (>14 years, *n* = 1253) in France, the mean consumption of fish and fishery food was 285 g/week, which results in mean exposure of 0.43 μg MeHg/kg bw/week (median = 0.30 μg/kg bw/week) and at the 97.5th percentile = 1.78 μg/kg bw/week [27].

The THg intake of the Catalonian population was calculated based on the consumption data of 2158 people and amounted to 0.78 μg/kg bw/week. These results were compared with PTWI THg (5 μg/kg of bw) [28]. Among 300 students of a middle secondary school in Sesimbra, Portugal [29] with the average number of fish meals consumed by each person 4.1 per week, the PTWI value applicable in Portugal (1.6 μg/kg bw) reached the value of 4.5. For the median of the Italian fish and seafood consumers, the EWI for a person weighing 65 kg amounted to 0.88 μg/week; it means 55% of PTWI (1.6 μg/kg bw) [30].

Tang et al. [31] have estimated exposure to THg and MeHg in secondary school students in Hong Kong (data obtained by means of a semi-quantitative food frequency questionnaire) from fish intake at median levels: THg = 0.5–0.6 μg/kg bw/week (10–12% PTWI, average consumer) and 1.6–1.9 μg/kg bw/week (32–38% PTWI, high consumer) and MeHg = 0.4–0.5 μg/kg bw/week (25–31% PTWI, average consumer) and 1.2–1.4 μg/kg bw/week (75–88% PTWI, high consumer). Authors compared their results with the PTWI value of THg equals 5 μg/kg bw/week set by the WHO in 1978 [1]. In another study from Hong Kong, Chen et al. [32] have shown that the average fish consumption (including seafood) for women in child-bearing age (20–49 years) amounted to 450 g/week. The high consumers ate 1500 g of fish and seafood per week. Their results indicate that the mean exposure to MeHg from the total diet was 0.68 μg MeHg/kg bw/week (age 20–29 years), 0.78 μg MeHg/kg bw/week (age 30–39 years), and 0.69 μg MeHg/kg bw/week (age 40–49 years). The 95th percentile was 2.1, 2.5, and 2.4 μg MeHg/kg bw/week, respectively. About 11% of those women had a dietary exposure to MeHg, exceeding the PTWI established by the JECFA.

For people from Japan, the world’s top fish and seafood consumers, Yaginuma-Sakurai et al. [33] have conducted an intervention study. It took 14 weeks. Twenty-seven volunteers (14 men and 13 women) ate only two species of fish—large predators with expected high levels of mercury, i.e., bigeye tuna and swordfish. In Japan, the PTWI value, which is safe for adults, amounts to 3.4 μg MeHg/kg bw/week. After the end of that, the observation continued for the next 15 weeks. During the experiment, the hair mercury levels were measured. Japanese people had baseline THg-H level of 2.30 ± 1.08 (microgram per gram). Contrary to their volunteers, our study subjects had baseline THg-H of 0.24 ± 0.16 μg/g. The authors compared our results with the PTWI value of THg 3.4 μg/kg bw/week recommended in 1973 by the Ministry of Health and Welfare of Japan.

Thapa et al. [34] have calculated MeHg intake (microgram per kilogram bw/week) based on fish consumption (kilogram per week) and Hg concentration in fish from Lake Phewa (Nepal) by different categories of people (*n* = 170). The minimum intake of MeHg (0.05 μg/kg bw/week) was found in the visitor (others) category, whereas hotel owners had the maximum intake (3.71 μg/kg bw/week). The minimum fish consumption for visitors (others) was 0.1 kg/week, and the maximum fish consumption for hotel owners was 7.5 kg/week. Authors have confirmed that MeHg intake per kilogram body weight depended on the species of fish being consumed. A person can consume 6.3 kg of tilapia, 3.5 kg of African catfish, and 2 kg of spiny eel weekly, and still the PTWI established by the FAO/WHO (1.6 μg/kg body weight) will not be exceeded. However, the same person can consume only 2.7, 1.5, and 0.9 kg of the mentioned fish species weekly to not exceed intake limit of 0.7 μg/kg bw/week set by the US-NCR.

## Conclusions

The estimated weekly intake in our research did not exceed the PTWI value suggested by the JECFA/ESFA for MeHg (1.6 μg/kg bw/week). However, 32.8% of the volunteers exceeded the PTWI values set by the US-NRC (0.7 μg/kg body weight). Nevertheless, when we took into consideration one species of fish with the highest amount of mercury, i.e., *M*. *magellanicus*, the EWI value exceeded PTWI among 13% individuals. Moreover, the results of average concentration of Hg in selected fish did not exceed the maximum permitted concentration (MPCs) for Hg in fish—0.5 mg/kg, established by the EU Commission Regulation (EC) [5]. The results indicate that the 67 volunteers of the research project would be unlikely to experience major toxicological effects of methylmercury.
